# Supplementary material for: The Role of the Insular Cortex and Serotonergic System in the Modulation of Long-Lasting Nociception
Source: Cells. 2024 Oct 17;13(20):1718. doi: 10.3390/cells13201718 (PMC11506361; doi:10.3390/cells13201718)
Supplement: Supplementary file 1 [file cells-13-01718-s001.zip › SF legends.pdf]

Supplementary Figure S1. An example of histological verification of the microdialysis cannula. An image from the Paxinos atlas overlapped with the slice. The figure shows the guide cannula (red arrow) and membrane trace (green arrow). The membrane location (green arrow) involves the three regions of the insular cortex (granular, GI; dysgranular, DI; and agranular, AI). The animals where off-site lesions were found were dropped out of the “n” in the corresponding group.

Supplementary Figure S2. Total paw volume (TPV, in ml) 3 hours after induction of inflammation in the different experimental groups.

Supplementary Figure S3. Protein levels of serotonin receptors are quantified in the insula cortex (IC) after induction of inflammation. (**Upper**) Western blots were performed with the protein lysates of IC dissected from the first batch of animals. (**lower**) Western blots were performed with the protein lysates of IC dissected from the second batch of animals. (**Left to the right**) WB corresponds to the loading control, GAPDH; 5HT1A, 5HT2A, and 5HT3, respectively. Blots include proteins of the control group, 3 and 24 hours post-induction of inflammation, respectively.
