# Supplementary figures and images for: The Role of the Insular Cortex and Serotonergic System in the Modulation of Long-Lasting Nociception
Source: Cells. 2024 Oct 17;13(20):1718. doi: 10.3390/cells13201718 (PMC11506361; doi:10.3390/cells13201718)

Figure 24

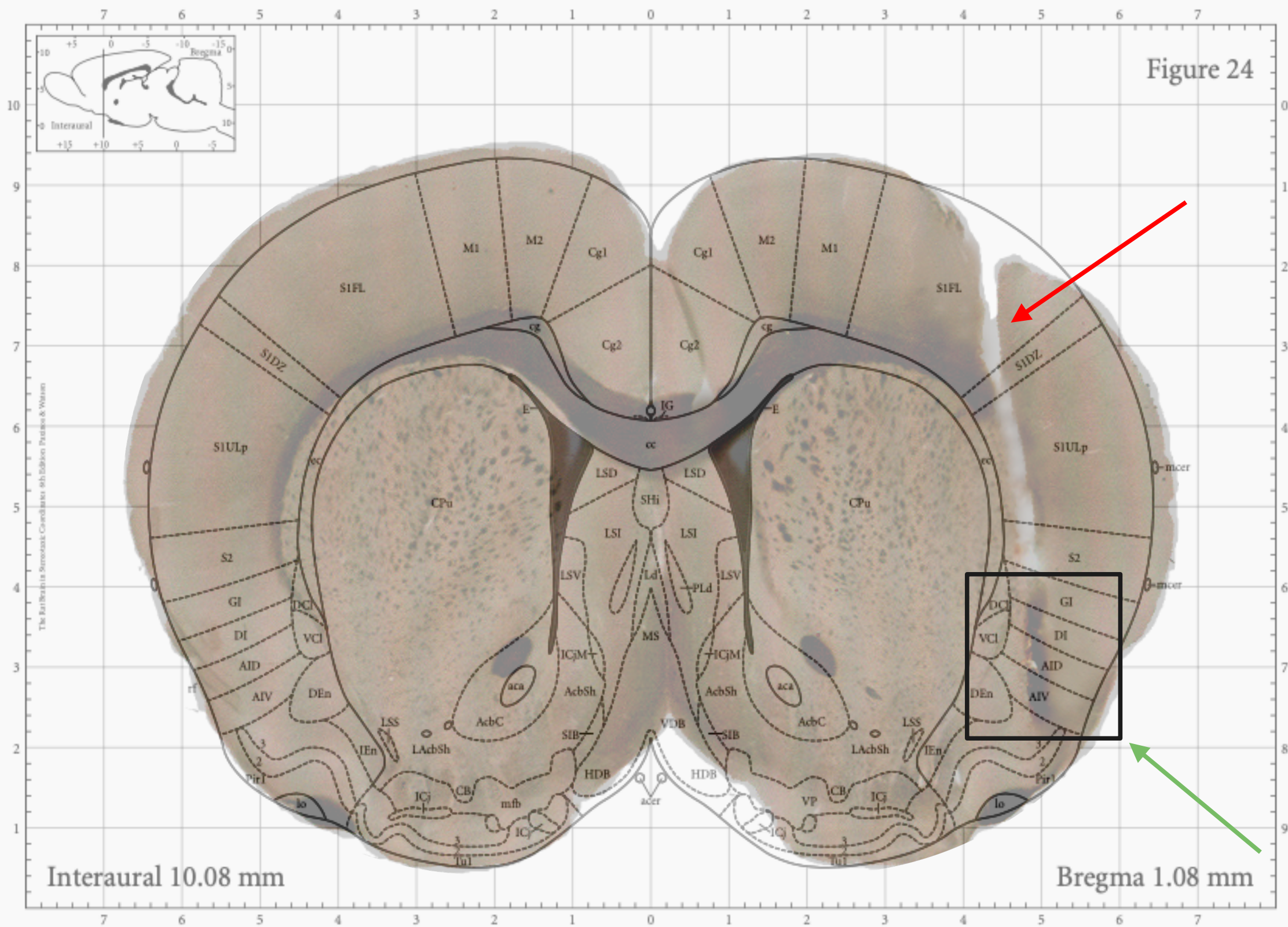

Supplement: Supplementary file 1 [file cells-13-01718-s001.zip › SF 1.pdf]

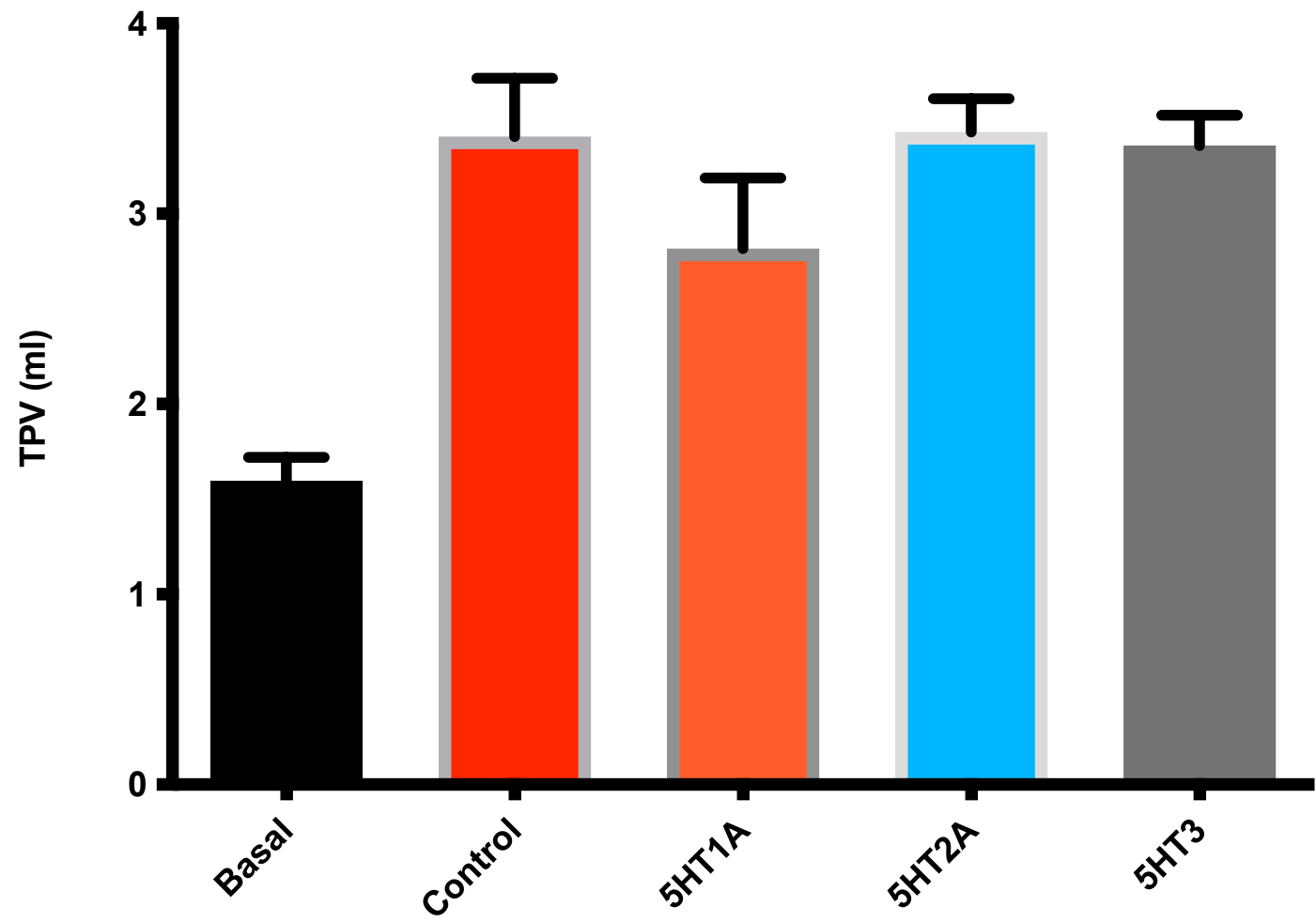

Supplement: Supplementary file 1 [file cells-13-01718-s001.zip › SF 2.pdf]

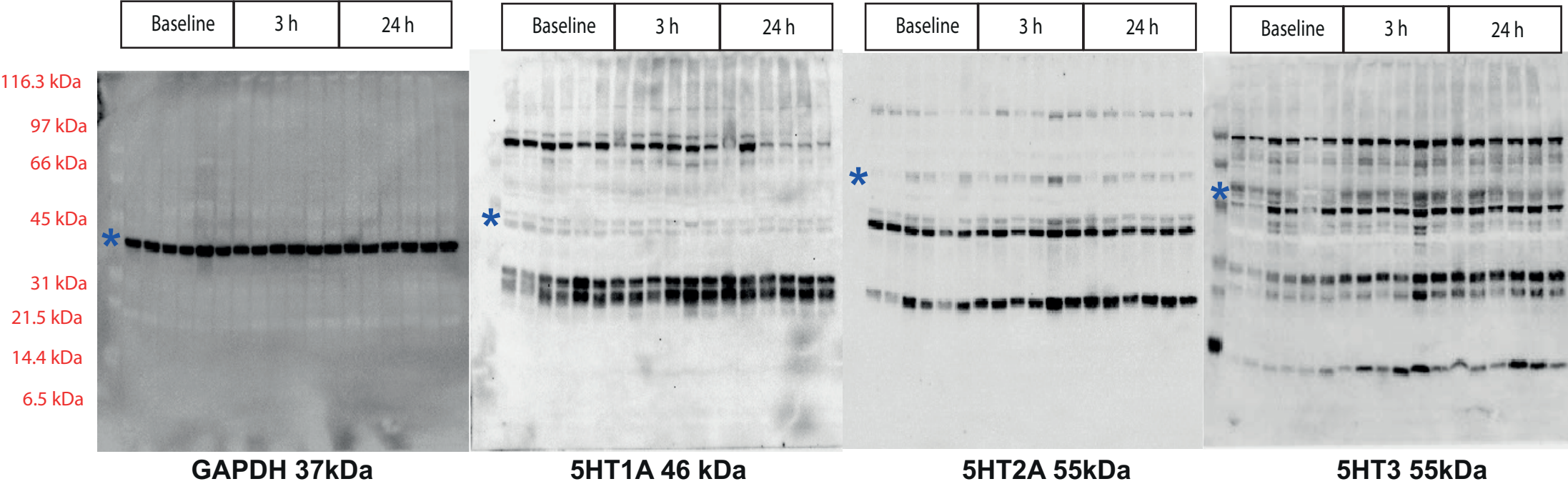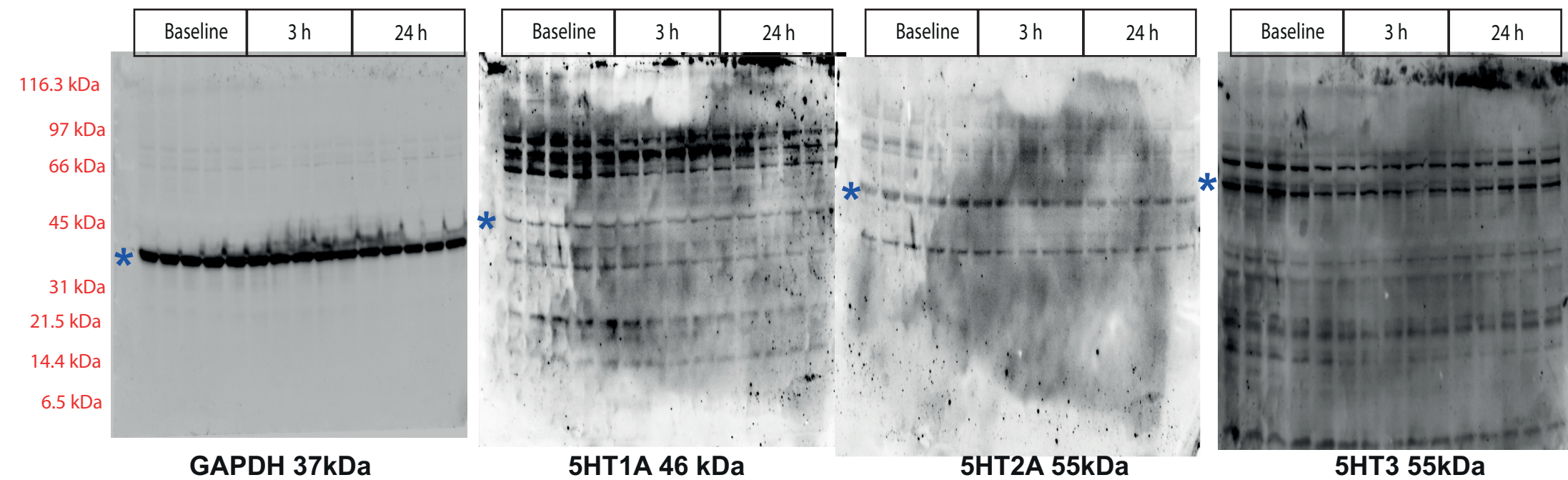

Supplement: Supplementary file 1 [file cells-13-01718-s001.zip › SF 3.pdf]
